# A novel antiangiogenic peptide derived from hepatocyte growth factor inhibits neovascularization in vitro and in vivo

**Published:** 2010-10-07

**Authors:** Yi Xu, Hui Zhao, Ying Zheng, Qing Gu, Jianxing Ma, Xun Xu

**Affiliations:** 1 Department of Ophthalmology, Shanghai First People’s Hospital, Shanghai JiaoTong University, Shanghai, China; 2 Department of Physiology, University of Oklahoma Health Sciences Center, Oklahoma City, OK

## Abstract

**Purpose:**

To study the antiangiogenic activity of two small peptides (H-RN and H-FT) derived from the hepatocyte growth factor kringle 1 domain (HGF K1) using in vitro and in vivo assays.

**Methods:**

RF/6A rhesus macaque choroid-retina endothelial cells were used for in vitro studies. The inhibiting effect of two peptides on a vascular endothelial growth factor (VEGF)-stimulated cell proliferation, cell migration, and endothelial cell tube formation were investigated. For in vivo assays, the antiangiogenic activity of H-RN and H-FT in the chick chorioallantoic membrane model (CAM) and a mice oxygen-induced retinopathy model (OIR) were studied. A recombinant mouse VEGF-neutralizing antibody, bevacizumab, and a scrambled peptide were used as two control groups in separate studies.

**Results:**

H-RN effectively inhibited VEGF-stimulated RF/6A cell proliferation, migration, and tube formation on Matrigel™, while H-FT did not. H-RN was also able to inhibit angiogenesis when applied to the CAM, and had antineovascularization activity in the retinal neovascularization of a mouse OIR model when administrated as an intravitreous injection. The antiangiogenic activity of H-RN was not as strong as that of VEGF antibodies. The H-FT and scrambled peptide had no such activity.

**Conclusions:**

H-RN, a new peptide derived from the HGF K1 domain, was shown to have antiangiogenic activity in vitro and in vivo. It may lead to new potential drug discoveries and the development of new treatments for pathological retinal angiogenesis.

## Introduction

There has been rapid growth in diabetes mellitus prevalence, and diabetic retinopathy (DR) has become the leading cause of blindness in adults of working age [1]. With the survival rate of premature infants increasing, retinopathy of prematurity (ROP) has become the major cause of blindness in children under the age of seven [2,3]. Both DR and ROP are likely to emerge as major public health threats in the near future. Neovascularization is the critical pathological process in both of these vascular retinopathies that cause blindness [4–7]. This pathological change is accompanied by retinal edema, blood-retinal barrier breakdown, hemorrhage, irreversible tissue damage, and scarring [8–14]. Laser photocoagulation is the current established therapy for retinal neovascularization and is effective in delaying the progression of the disease. However, it lacks specificity, and causes retinal destruction, impaired visual function, and scotoma [15]. Photodynamic therapy, which is a relatively newly developed treatment, blocks existing neovasularization but does not prevent pathological angiogenesis. Treatments that selectively inhibit or block the molecular mediators of neovascularization are needed [16].

Vascular endothelial growth factor (VEGF) is a major mediator of the angiogenic process. Numerous therapeutic strategies targeting VEGF are being studied [16,17]. New therapeutic agents, such as pegaptanib, ranibizumab, and bevacizumab, have been developed. Some of them have been approved by the US Food and Drug Administration, and the others are still in clinical trials [16]. The extremely high annual drug-treatment cost, however, has limited their wide use, and systemic adverse events have been reported. For instance, intravenous bevacizumab can cause hypertension, congestive heart failure, bleeding, neutropenia, proteinuria, thromboembolism, and neuropathy [18,19]. Though intravitreal application might circumvent these adverse effects, breakdown of the tight blood-ocular barrier is common in neovascular eye diseases [20], and systemic exposure is inevitable. Other well known angiogenic inhibitors, such as angiostatin, endostatin, and thrombospondin-1, are large, complex proteins and are therefore difficult and costly to manufacture [21].

Designing and developing peptides to inhibit angiogenesis is an important area of antiangiogenic drug development [21]. Compared to proteins, peptides have lower immunogenicity, higher solubility in water, stable production methods, and improved consistency between batches [16,21]. They are also better at targeting and penetrating tumors [22]. Endogenous protein angiogenesis stimulators and inhibitors have been considered, and there is plenty of information available for designing antiangiogenic peptides for drug development [23–28]. Conserved protein domains carry phylogenetic information, have structural roles, and perform unique functions; hence, they have always been an important source for designing antiangiogenic peptides [21,25,29]. Antiangiogenic peptides derived from thrombospondin [30], endostatin [31], decorin [32], tumstatin [33], and histidine-proline-rich glycoprotein [34], for example, have been widely reported, and some peptide drugs with antiangiogenic treatment purposes have entered clinical trials.

Hepatocyte growth factor (HGF) was first discovered to strongly promote liver cells growth [35]. Subsequently, HGF was found to play a significant biologic role in health and disease [36,37]. HGF is a potent stimulator of new vessel formation and an important angiogenic factor in vascular retinopathies, such as proliferative DR and ROP [38–42]. The mature form of HGF consists of disulphide-linked α- and β-chains. The α-chain is made up of an N-terminal domain and four kringle domains (an 80-amino acid triple-loop structure maintained by three intramolecular disulfide bonds highly conserved between different kringle-containing proteins [43]). The β-chain consists of a single domain that retains the fold of the catalytically active serine proteinases but has no enzymatic activity [44]. Though wild-type HGF is itself a strong angiogenic stimulator, several HGF variants have been constructed and have been shown to have antiangiogenic activity, such as NK4 (composed of the NH2-terminal hairpin domain and four kringle domains in the α-chain of HGF) [45,46], kringle 1–4 [47], the N-terminal domain [48], and kringle 1 [49–51]. Xin et al. [49] first discovered that the kringle 1 domain of HGF (HGF K1) inhibited endothelial cell proliferation stimulated by basic fibroblast growth factor, and that it caused cell apoptosis. It was shown to be three times stronger than angiostatin in inhibiting the proliferation of endothelial cells. HGF K1 was then reported to exhibit both antiangiogenic and antitumor cell effects [50,51].

Angiogenesis is the formation of new blood vessels from the preexisting vasculature. This process depends on the proliferation, migration, and capillary-like tube formation of vascular endothelial cells [52]. The HGF K1 domain has been shown to be an angiogenesis inhibitor. We chose two conserved sequence segments of the HGF K1 domain to construct two small peptides, using a solid-phase peptide synthesis method [53]. To address the issue of whether the two small peptides have antiangiogenic activity, we performed in vitro assays on the choroid-retina endothelial cell line (RF/6A) and in vivo assays for angiogenesis in the chorioallantoic membrane (CAM) model and in mice. We discovered that one of the two peptides inhibited RF/6A cell proliferation, migration, capillary-like tube formation, and angiogenesis in the CAM model and in a mouse ROP model (OIR), while the other peptide did not. The newly discovered peptide with antiangiogenic activity could likely serve as a therapeutic peptide drug in vascular retinopathies and other angiogenic-related diseases, such as tumors.

## Methods

### Cell culture and materials

RF/6A cells were obtained from the American Type Culture Collection and were maintained as monolayer cultures in a culture medium consisting of RPMI1640 supplemented with 10% fetal bovine serum (Gibco, Rockville, MD) at 37 °C in 5% CO_2_ and 95% ambient air. The culture medium was changed every 3 days. Human VEGF165 was obtained from (Sigma-Aldrich, St. Louis, MO). Two conserved amino acid sequences of HGF K1 (H-RN: RNPRGEEGGPW, molecular weight: 1254.34 Da; H-FT: FTSNPEVRYEV, molecular weight: 1340.47 Da) and a scrambled peptide were synthesized by solid-phase peptide synthesis using an automatic peptide synthesizer (Symphony; Protein Technologies, Tucson, AZ). The end product was characterized by high-performance liquid chromatography (HPLC; LC-20A, Shimadzu, Kyoto, Japan) and mass spectrometry (MS; Finnigan TSQ 7000; Thermo, Waltham, MA). Both of the synthesized peptides were water-soluble.

### Stability assay of peptides in aqueous solutions

The stability of peptides in various aqueous solutions was investigated, as previously described [54], with slight modification. Briefly, lyophilized H-RN, H-FT and scrambled peptide were dissolved in water or in buffer at a concentration of 200 µg/ml. Buffers included: a) 12.5 mM citrate and 14 mM NaCl pH 6; b) 12.5 mM citrate and 46.2 mM NaCl pH 4; c) Hanks balanced salt solution (HBSS) pH 6; d) HBSS pH 7.4; e) phosphate buffered saline (PBS, 0.137 M NaCl, 0.0027M KCl, 0.01M Na_2_HPO_4_, 0.002 M KH_2_PO_4_) pH 7.4; and f) BSS PLUS™ sterile intraocular irrigating solution (Alcon, Inc.; Hünenberg, Switzerland). BSS PLUS™ is an intraocular irrigating solution for use during all intraocular surgical procedures (used to imitate the intraocular condition). The peptide solutions were incubated at 4 °C or 37 °C for 4, 24, or 48 h, then stored at −80 °C. The solutions without incubation and immediately frozen at −80 °C were used as controls. The stability of peptides was evaluated by HPLC. Samples were thawed at room temperature for 5 min before testing.

### Cell proliferation assay

A cell proliferation assay was performed as described previously [55,56]. A colorimetric MTS (3-(4,5-dimeth-ylthiazol-2-yl)-5-(3-carboxymethoxyphenol)-2-(4-sulphophenyl)-2H-tetrazolium) assay (CellTiter 96 AQ; Promega, Madison, WI) was used according to the manufacturer’s instructions. Briefly, cells were seeded onto a 96-well plate (4.8×10^3^ cells/well). After 24 h, cells were serum-starved overnight, and then treated with 100 ng/ml VEGF and various doses of H-RN or H-FT (0, 1 μM, 10 μM, 100 μM, or 1 mM) in 100 μl of serum-free medium for an additional 24 h. In positive control group, 100 μl of serum-free medium contains 100 ng/ml VEGF and 0.25 or 2.5 mg/ml bevacizumab (Avastin®, Hoffmann-La Roche Ltd., Basel, Switzerland), and the negative control group contains 100 ng/ml VEGF and a scrambled peptide at concentrations of 1 μM, 10 μM, 100 μM, or 1 mM. After completion of the treatment, 20 μl of the 3-(4,5-dimethylthiazol-2-yl)-5-(3-carboxymethoxyphenol)-2-(4-sulphophenyl)-2H-tetrazolium (MTS) reagent were pipetted into each well and incubated for 4 h. The absorbance at 490 nm was recorded using a microplate reader (Bio-Rad, Model 680, Hercules, CA). Each group was tested at least in triplicate, and the assays were repeated a minimum of three times.

### Cell migration assay

A cell migration assay was performed as described previously [57], with modification as below. Briefly, RF/6A cells were starved overnight, trypsinized, and suspended at a final concentration of 5×10^5^ cells/ml. Various concentrations of bevacizumab or peptides were preincubated with the cells for 30 min at 37 °C before seeding into the Transwell chambers (pretreated by culture medium, 10 mm diameter, 8.0 µm pore size; Corning Inc., New York, NY), the 5×10^4^ cells were added to each upper chamber. VEGF (100 ng/ml) was placed into the lower chamber. The assembled cell-culture insert chamber was then incubated at 37 °C for 24 h. After removing the nonmigrating cells in the upper chambers with a cotton swab, migrated cells on the lower surface of the porous membrane were fixed, stained with Gram stain, and photographed under a light microscope (Olympus, Tokyo, Japan). Five random fields (×200) were chosen in each insert, and the cell number was quantified manually. Each experiment was repeated three times.

### Endothelial cell tube formation assay

A tube formation assay was performed as previously described [58]. The 96 well culture plate were coated with Matrigel™ (BD Biosciences, Bedford, MA) according to the manufacturer’s instructions. RF/6A cells (2.5×10^4^ in number) were preincubated with various concentrations of bevacizumab or peptides (100 nM–1 mM) before being seeded on coated plates in a serum-free medium containing VEGF 100 ng/ml, and then incubated at 37 °C for 6 h. Tube formation was observed using an inverted phase contrast microscope (Olympus, Tokyo, Japan). Images were captured with a digital camera (Olympus). The degree of tube formation was quantified by measuring the length of tubes in three randomly chosen fields (with magnification of ×100) from each well using the Image-Pro Plus Program (version 5.1; Media Cybernetics, Inc.). Each group was tested in triplicate. Each experiment was repeated three times.

### Chick chorioallantoic membrane assay

A CAM assay was performed to determine the antiangiogenic activity of the peptides. Briefly, 5 μl of 0.01 M PBS (0.137 M NaCl, 0.0027M KCl, 0.01M Na2HPO4, 0.002 M KH2PO4, pH 7.4) containing H-RN, H-FT, or scrambled peptide separately at concentrations of 0, 2, or 10 μg/μl were loaded onto a 0.5 cm-diameter Whatman quantitative ﬁlter paper (Sigma-Aldrich), and the ﬁlter was dried under sterile air. The ﬁlter was then applied to the CAM of an eight-day embryo. After incubation for 72 h, angiogenesis around the test materials was microscopically evaluated using an Olympus SZX2-ILLT stereoscope. The number of blood vessels was quantified manually in a circular perimeter surrounding the implants, at a distance of 0.25 cm from the edge of the filter. Assays for each test sample were performed twice, and each experiment contained 10–15 eggs per data point [59]. All analyses were done with the observer masked as to the study group.

### Animal model of proliferative retinopathy

All experiments were consistent with the ARVO Statement for the Use of Animals in Ophthalmic and Vision Research. A reproducible model of ischemia-induced retinal neovascularization has been described in detail [60]. Pregnant female C57BL/6J mice were obtained from the Shanghai Laboratory Animal Center, Chinese Academy of Sciences. Each litter contained 7–9 pups; each group was consisted of 7–9 pups. Briefly, on postnatal day 7 (P7), litters of C57BL/6J mouse pups with their mothers were exposed to 75%±2% oxygen (hyperoxia) for 5 days and then returned to room air for 5 days, producing retinal ischemia and neovascularization by P17. Mice of the same age were kept in room air and used as normal control subjects. Peptides were administered as intravitreous injections at concentrations of 10 mM or 50 mM. PBS and anti-mouse VEGF antibodies (VEGFab, a neutralizing antibody to VEGF that recognizes the mouse; Sigma-Aldrich) were injected as controls. PBS and peptide injections were performed twice on P12 and P14, and a VEGFab injection was given once on P12, according to Geisen et al. [14]. For the intravitreous injections, a topical anesthetic (0.4% oxybuprocaine, Santen Pharmaceutical Co., Ltd., Hakui-gun, Ishikawa, Japan) was administered before inserting a sterile 30-gauge needle posterior to the limbus to avoid lens damage. A 1 µl injection was performed in the left eyes using a Hamilton syringe. Topical Ofloxacin ointment (0.3%; Santen Pharmaceutical Co., Ltd.) was then applied to the injected eye. All the right eyes were not injected. Pups were then returned to their mothers in room air for 5 days [14].

At P17, retinal tissue was dissected and flatted, and eye sections were performed. In detail, the pups were deeply anesthetized intraperitoneally with tribromoethanol (0.2 ml/10 g bodyweight) and then perfused through the left ventricle with 4% paraformaldehyde. Both eyes were enucleated and fixed in 4% paraformaldehyde for 2 h. The retinas were dissected and flattened. The retinal vasculature was stained with Alexa Fluor 568 conjugated isolectin B_4_ (Molecular Probes, Eugene, OR) as previously described [14]. Briefly, the dissected retinas were permeabilized in ice-cold 70% ethanol for 20 min, then in 1% Triton X−100 for 30 min, and then incubated with 5 μg/ml isolectin B_4_ overnight at 4 °C. Each retina was rinsed three times in PBS, mounted in PBS:glycerol (2:1) and protected with a coverslip. Isolectin B_4_ selectively binds with terminal α-D-galactosyl residues, and has strong affinity for perivascular cells and endothelial cells. Images of the retinal blood vessels were captured using an Olympus BX51 upright microscope. For sections, the eyes were embedded in paraffin after being fixed with 4% paraformaldehyde. Serial sections (10 μm) of whole eyes were cut sagittally, through the cornea and parallel to the optic nerve, and stained with hematoxylin and eosin. Vascular lumens, identified under light microscopy, were considered to be associated with new vessels if they were found on the vitreal side of the internal limiting membrane. New vessel lumens were counted in cross-section with light microscopy (magnification, 400×) by two masked observers [14]. Each data point represented a minimum of one eye each from seven pups. The efﬁcacy of treatment was qualitatively evaluated by isolectin B_4_-stained flat mounting retinal tissue, and was quantitatively evaluated by comparison of the average number of neovascular lumens per section in the eyes of peptide-treated animals and controls [8].

### Statistical analysis

For experiments with more than two treatment groups and various treatment concentrations, univariate ANOVA was used. For comparison of the differences between groups, a post hoc Least-Significance-Difference (LSD) test was used. All values were expressed as the mean±SD. An alpha level of <0.05 was used as the criterion of significance.

## Results

### Stability of H-RN, H-FT, and scrambled peptide

H-RN, H-FT, and scrambled peptide were tested in different aqueous solutions at different temperatures and storage times. The stability was analyzed as a percentage of the purity of control solutions. Details are shown in Table 1. Three peptides were less stable in PBS (pH 7.4, 37 °C), citrate buffer (pH 6, 37 °C), and BSS PLUS™ (pH 7.4, 37 °C). Some peptide degradation happened after incubation for 24–48 h. However, the degradation only happened in a small part of the total peptide content, and more than 90% still existed after 48 h. The three peptides had high stability under the other six incubation conditions. From the data in Table 1, we inferred that 4 °C and an acidic pH are optimal storage conditions for the higher stability of these peptides.

**Table 1:** Stability of H-RN, H-FT and scrambled peptide in different conditions

				**Mean±SD percentage of controls**		
**Medium**	**pH**	**Temperature (°C)**	**Time (h)**	**H-RN**	**H-FT**	**Scrambled peptide**
Citrate buffer	6	4	4	99.97±0.02	98.99±0.36	99.85±0.18
			24	99.83±0.19	99.65±0.18	100.02±0.06
			48	99.29±0.31	98.01±0.23	99.73±0.20
PBS	7.4	4	4	99.91±0.37	99.96±0.17	100.03±0.07
			24	99.82±0.39	99.12±0.05	99.68±0.11
			48	100.18±0.31	98.89±0.22	99.65±0.12
PBS	7.4	37	4	100.1±0.23	99.69±0.08	100.12±0.18
			24	99.44±0.09*	98.56±0.12*	99.25±0.16*
			48	97.89±0.48*	98.09±0.13*	98.29±0.29*
H_2_O	4.7	37	4	99.84±0.04	99.62±0.21	99.43±0.06
			24	99.99±0.06	99.53±0.08	99.32±0.05
			48	99.78±0.19	99.25±0.11	99.28±0.16
Citrate buffer	4	37	4	99.57±0.57	99.42±0.27	100.06±0.05
			24	99.85±0.40	99.35±0.19	99.72±0.13
			48	99.59±0.11	99.13±0.25	99.28±0.21
Citrate buffer	6	37	4	98.97±0.03*	99.86±0.11	99.54±0.15
			24	97.98±0.03*	98.04±0.23*	98.02±0.33*
			48	94.71±0.08*	97.88±0.09*	98.78±0.19*
HBSS	6	37	4	98.82±1.24	100.06±0.08	99.73±0.18
			24	100.15±0.11	99.18±0.23	99.64±0.06
			48	98.60±0.08	99.65±0.09	99.23±0.27
HBSS	7.4	37	4	100.20±0.11	99.93±0.24	99.86±0.15
			24	99.90±0.22	99.67±0.18	100.02±0.07
			48	99.28±0.63	99.25±0.09	99.37±0.21
BSS PLUS™	7.4	37	4	99.85±0.25	98.61±1.28	98.81±0.35
			24	99.95±0.05	95.22±2.09*	97.44±0.29*
			48	98.12±0.26*	97.53±0.32*	98.01±0.18*

No statistical differences unless otherwise stated. ^#^ Standard deviation; * p<0.05 (tested condition compared to control).

### RF/6A cell proliferation inhibition by H-RN rather than H-FT

**Figure 1:**
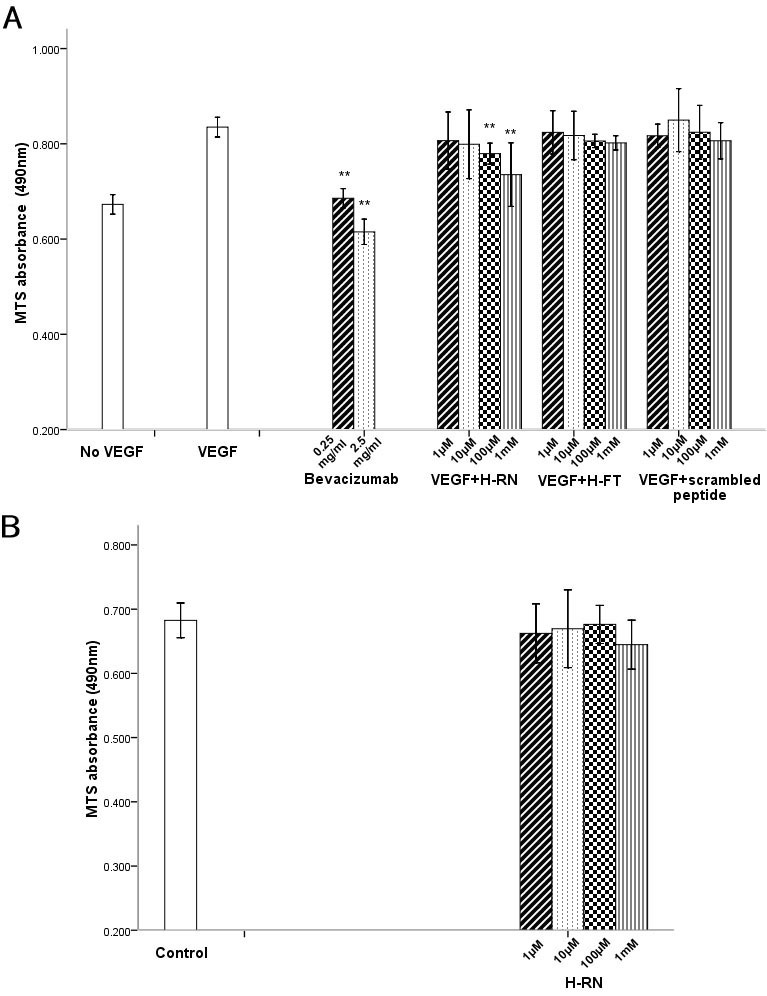
H-RN inhibits endothelial cells (EC) proliferation while H-FT does not. **A**: After 24 h of starvation, rhesus macaque choroid-retina endothelial (RF/6A) cells were incubated in vascular endothelial growth factor (VEGF) and various concentrations of bevacizumab or peptides for 24 h; RF/6A cell proliferation was inhibited by bevacizumab and H-RN. An H-RN concentration of 1 mM inhibited the VEGF-induced response significantly. However, the inhibitory effect was not as strong as bevacizumab’s. H-FT and scrambled peptide had no inhibitory activity on RF/6A cells at various concentrations (**p<0.01, each condition versus control). **B**: Compared to the culture medium group, cell viability was not affected by H-RN alone at any concentration tested (p>0.05, each condition versus control.)

RF/6A cells were exposed for 24 h to VEGF (100 ng/ml), or to VEGF combined with various concentrations of bevacizumab, H-RN, or H-FT. In comparison to the control group, VEGF alone induced an increase of cell proliferation (p<0.05; Figure 1). VEGF-induced cell proliferation was strongly inhibited by 2.5 mg/ml bevacizumab. After adding different concentrations of peptides, H-RN resulted in a significant reduction in cells (p<0.05). At the highest dose of H-RN tested (1 mM), the mean absorbance value was very close to that of the control group (0.735 versus 0.673), though this inhibitory effect was not as strong as that of 2.5 mg/ml bevacizumab (0.735 versus 0.615). In contrast, in the H-FT groups, no inhibitory effect on cell proliferation was observed, even at a millimolar concentration (p>0.05). The scrambled peptide group showed no inhibitory effect, either (p>0.05; Figure 1A). These data indicate that increasing H-RN concentration may be responsible for the impaired RF/6A cell proliferation in response to VEGF165; H-FT could not inhibit RF/6A cell proliferation. Another group treated with H-RN alone was tested to detect whether the high dose of H-RN might induce cell death instead of inhibition of cell proliferation. As can be observed in Figure 1B, cell viability was not affected by H-RN at any concentration tested, as compared to the culture medium group (control).

### H-RN suppression of VEGF-driven migration

**Figure 2:**
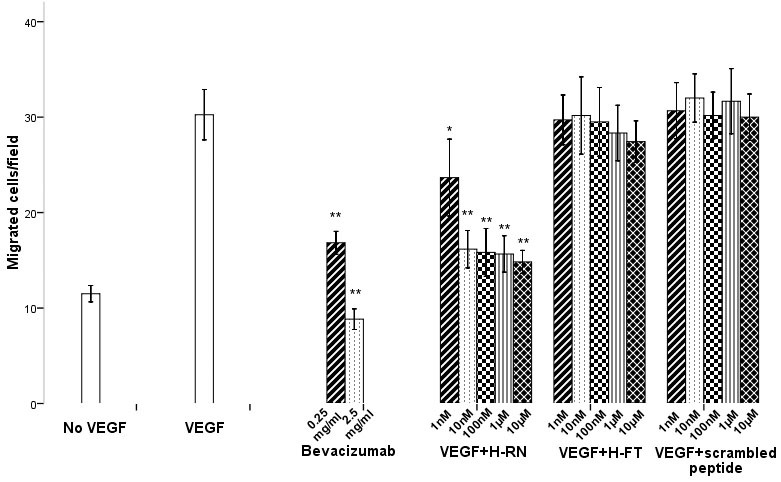
Effects of peptides on the migration of RF/6A cells stimulated by vascular endothelial growth factor. Bevacizumab and peptides were added in the upper chamber containing RF/6A cells 30 min before the stimulation of vascular endothelial growth factor (VEGF) in the lower chamber (100 ng/ml). After 24 h of incubation, migratory activity of the cells was estimated. Bevacizumab and H-RN inhibited migration of RF/6A cells. H-FT and scrambled peptide did not inhibit migration up to 1 µM concentration, whereas at 10 µM there was a mild inhibition (*p<0.05; **p<0.01, each condition versus control).

The migration of RF/6A cells was studied using a Transwell chamber method by which the endothelial cells migrated through a porous membrane toward a VEGF stimulus. The inhibitory activity of various concentrations of H-RN and H-FT (1 nM, 10 nM, 100 nM, 1 µM, and 10 µM) on endothelial cell migration was studied. Bevacizumab and scrambled peptide were also added as controls. Compared to the no-VEGF group, the number of migrated cells in the VEGF group was significantly higher (p<0.001). The results showed that bevacizumab effectively inhibited cell migration at both concentrations (p<0.001). H-RN inhibited VEGF-stimulated endothelial cell migration with an ED_50_ around 10 nM, whereas H-FT did not inhibit VEGF-induced cell migration at concentrations from 1 nM to 1 µM. H-FT had only a mild inhibitory effect at the 10 µM concentration, and this observation was not statistically significant (p>0.05; Figure 2). The scrambled peptide did not present any inhibitory effects.

### H-RN and H-FT mediation of different inhibitory effects on endothelium tube formation

**Figure 3:**
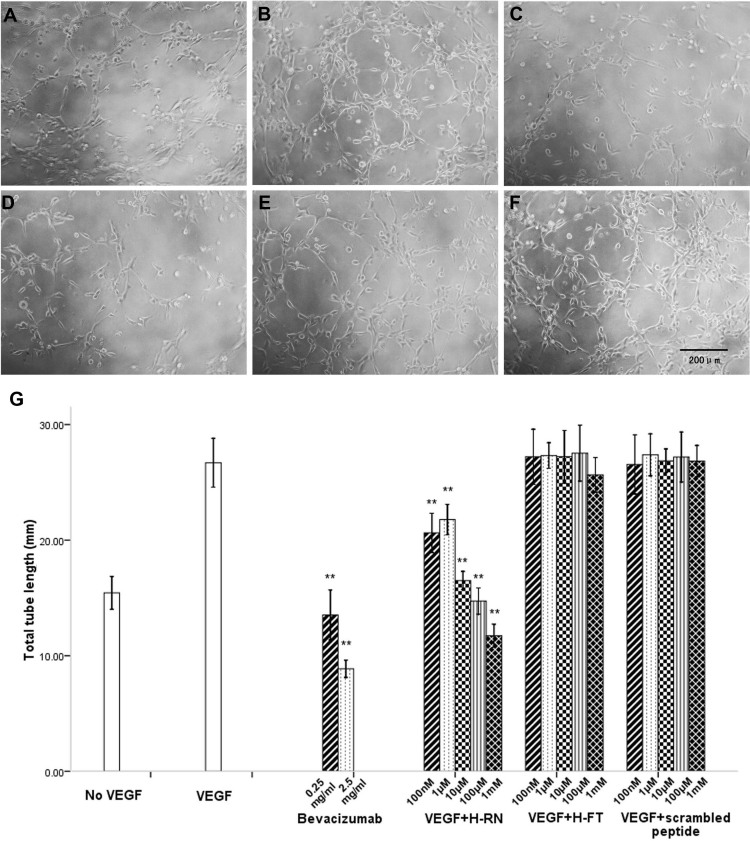
Inhibition effects of H-RN and H-FT on endothelial cell differentiation into capillary structures. Starved RF/6A cells were pre-incubated with different concentrations of bevacizumab, H-RN, and H-FT for 30 min before 100 ng/ml vascular endothelial growth factor (VEGF) was added in peptides treated and VEGF groups, and then cells were seeded into Matrigel-coated wells. **A-F**: Representative photographs of anti-tube formation activity of bevacizumab, H-RN, H-FT, and scrambled peptide: **A**, no-VEGF; **B**, 100 ng/ml VEGF; **C**, 100 ng/ml VEGF +2.5 mg/ml bevacizumab; **D**, 100 ng/ml VEGF +1 mM H-RN; **E**, 100 ng/ml VEGF +100 nM H-FT; **F**, 100 ng/ml VEGF +1 mM scrambled peptide. VEGF at 100 ng/ml strongly stimulated endothelium tube formation. Bevacizumab and H-RN potently inhibited VEGF-stimulated RF/6A cell tube formation on growth factor-reduced Matrigel™. H-FT and scrambled peptide did not inhibit RF/6A cell tube formation effectively (magnification ×200). **G**: Quantitative analysis of tube formation under the different experimental conditions using Image Program Plus software. The values are mean tube lengths from three repeated experiments (** p<0.01, each condition versus control).

The amount of 100 ng/ml of VEGF strongly stimulated RF/6A cell endothelium tube formation, compared to the no-VEGF group. Tube formation was strongly inhibited by bevacizumab at both concentrations. Similarly, but not as strongly as with bevacizumab, H-RN inhibited VEGF-induced RF/6A cell tube formation on Matrigel™ much more potently than did H-FT (Figure 3A-F). H-RN effectively inhibited tube formation (p<0.001), and its ED_50_ was around 100 µM. In contrast, H-FT did not effectively inhibit VEGF-induced RF/6A cell tube formation (p>0.05; Figure 3G). It is most interesting that H-FT, at a 1 mM concentration, had a mild inhibitory effect on tube formation, though it appeared inactive in the cell proliferation assay. When experiments were repeated, consistent results were observed. The scrambled peptide did not inhibit VEGF-induced endothelium tube formation at any concentration (p>0.05).

### H-RN inhibition of chick chorioallantoic membrane angiogenesis

**Figure 4:**
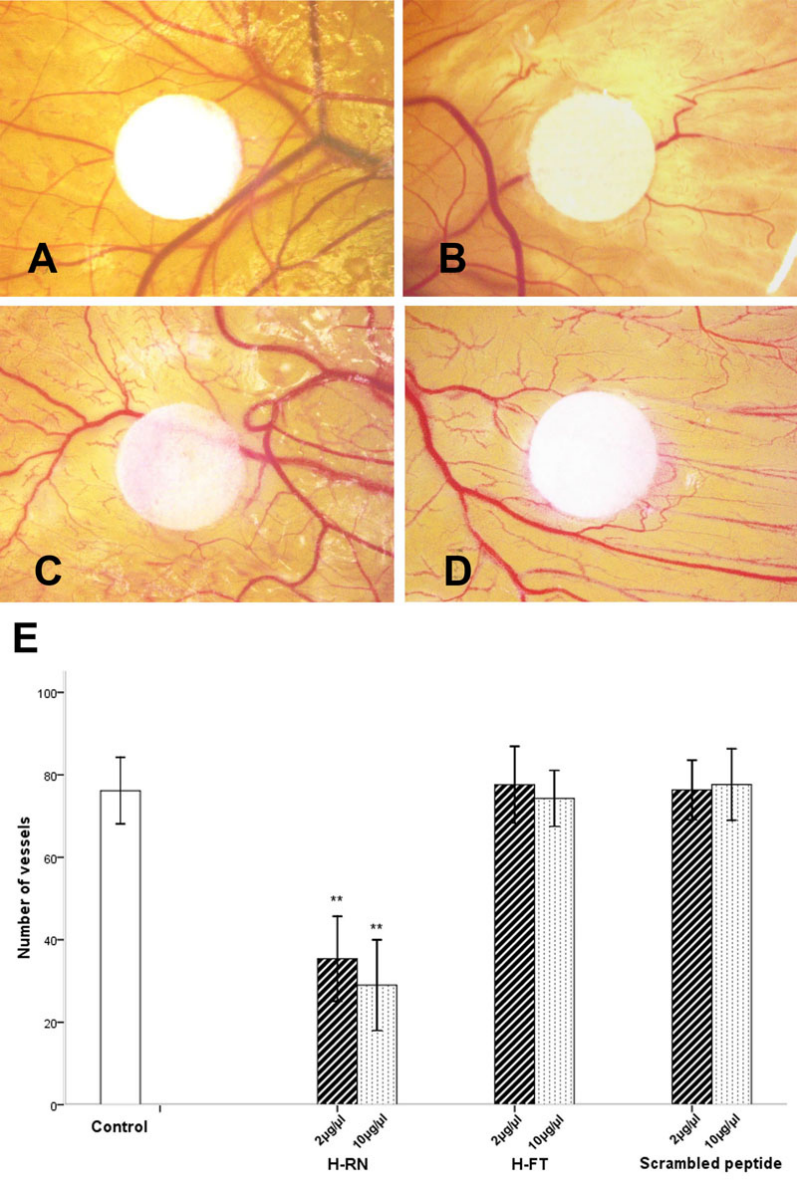
H-RN inhibits angiogenesis of chorioallantoic membrane model. **A-D**: Representative photographs of antiangiogenesis in chick chorioallantoic membrane (CAM) of H-RN, H-FT, and scrambled peptide. Filter paper disks saturated with H-RN (10 µg, 50 µg), H-FT (10 µg, 50 µg), scrambled peptide (10 µg, 50 µg) or PBS were placed on the CAMs for 72 h of incubation. Vessels around the filter disks were photographed: **A**, Control (PBS); **B**, 50 µg H-RN per egg; **C**, 50 µg H-FT per egg; **D**, 50 µg scrambled peptide per egg. (magnification was ×11.5). **E**: Quantitative results showed different effects of two peptides on CAM angiogenesis. H-RN inhibited CAM angiogenesis significantly, compared to the PBS group. No such antiangiogenesis activity of H-FT or scrambled peptide was found (** p<0.01, each condition versus control).

The CAM results indicated H-RN inhibited angiogenesis on CAM (Figure 4). In the 10 µg and 50 µg H-RN groups, the number of vessels decreased notably compared to PBS group 4,285 ogenesis on CAM (p<0.001). However, the H-FT and scrambled peptide presented no such activity at any concentration (p>0.05; Figure 4). The results demonstrate that H-RN was able to suppress angiogenesis in chicken embryos.

### Effect on retinal neovascularization of peptides administration

**Figure 5:**
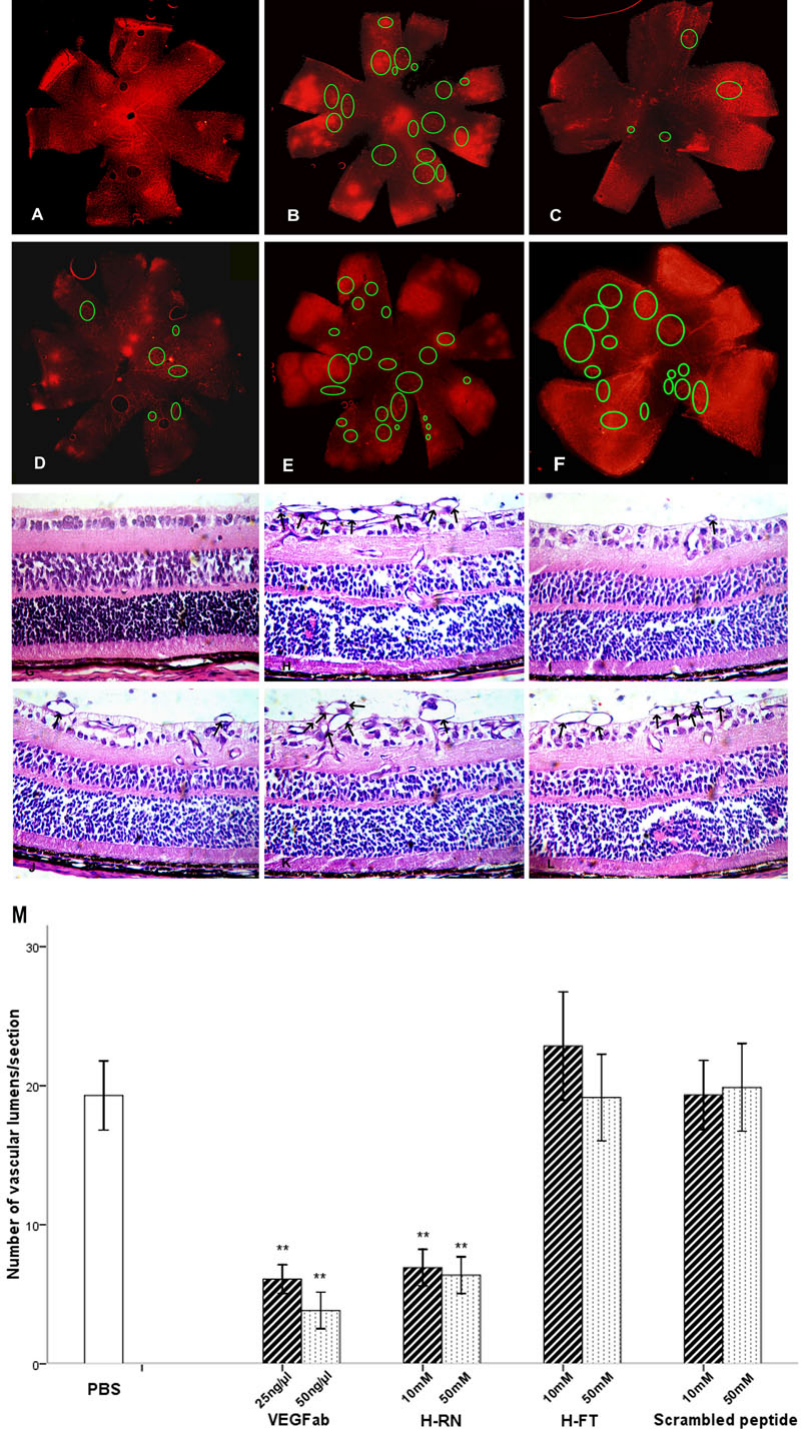
H-RN inhibits retinal neovascularization. **A**: Retinal vascular distribution of a normal pup raised in room air. **B**: This figure shows a representative retina from 17-day-old pups that were subjected to 5 days of 75% oxygen tension and then maintained in room air for an additional 5 days. Circles indicate the neovascular tufts. **C**: A retina from a mouse pup injected with vascular endothelial growth factor ab (VEGFab; 50 ng/µl). **D**: A retina from a mouse pup injected with H-RN (50 mM). **E**: A retina from a mouse pup injected with H-FT (50 mM). **F:** A retina from a mouse pup injected with scrambled peptide (50 mM, magnification, ×40). **G**: A hematoxylin- and eosin-stained section of P17 control raised in room air. **H:** P17 retina exposed to hyperoxia from P7 to P12, and returned to room air from P12 to P17. Arrows indicate neovascular tufts extending into the vitreous. **I**: A section from 50 ng/µl VEGFab-treated hyperoxia group. **J**: A section from 50 mM H-RN-treated hyperoxia group. The retina of **I** and **J** presented obvious reduced abnormal neovascularization tufts (arrows). **K**: A section from 50 mM H-FT-treated hyperoxia group. **L**: A section from 50 mM scrambled peptide treated hyperoxia group (magnification, ×400). **M**: Quantification of neovascular lumens response to hyperoxia. Lumens extending from the internal limiting membrane into the vitreous were counted. Data in each column present means±SD values of total number of vascular lumens per retinal cross-section, from 7 to 9 eyes of 7–9 mice. Note that the lumens in VEGFab and H-RN-treated group are reduced significantly more than the control and PBS groups (p<0.001), while the number of lumens in the H-FT and scrambled peptide-treated group had no difference from the control and PBS treated group (p>0.05). These experiments were repeated three times with similar results (** p<0.01, each condition versus control).

For qualitative analysis, retinal ﬂatmounts were prepared. The vasculature labeled by Alexa Fluor 568-conjugated isolectin B_4_ showed that there was no effect of H-FT on the pathologic neovascular tufts of the retinas of oxygen-treated mouse pups. In contrast, pathologic retinal neovascularization was signiﬁcantly inhibited by injection of VEGFab (at doses of 25 ng/μl or 50 ng/μl) and H-RN (both 10 mM and 50 mM; Figure 5A-F). The neovascular lumens were deﬁned as the mean number per section found in at least 10 sections per eye. Treatment with 25 ng/µl or 50 ng/µl VEGFab resulted in a significant reduction in preretinal lumens. Though not as remarkable as with VEGFab, injection of 50 mM H-RN still reduced neovascularization more than 60%, compared to the PBS control. Neither H-FT nor scrambled peptide showed significant reduction of preretinal lumens when compared to the PBS-injected eye (Figures 5G-M).

Taken together, these results suggest that H-RN can effectively inhibit pathologic retinal neovascularization in a mouse model of oxygen-induced retinopathy.

## Discussion

HGF is a polypeptide structurally related to the enzymes of the blood coagulation cascade, which has no antiangiogenic activity, but is a growth factor with angiogenic activity [48]. However, HGF K1 has been shown to be able to suppress tumor angiogenesis in hepatocyte cancer and colon cancer [50,51]. Due to peptides’ small size, simplicity of synthesis, lack of toxicity, immune reaction to the host system, and receptivity to chemical modification, they have been widely developed for the medication of tumors and other angiogenesis-related diseases. In this study, we synthesized two peptides derived from a conserved sequence of HGF K1 to investigate their potential antiangiogenic activity. It is well known that VEGF is often viewed as the gold standard of angiogenic factors. It is the most important angiogenic factor to induce endothelial cell proliferation, migration, and tube formation [61]. This is the reason we chose VEGF as the negative control in the in vitro studies.

Bevacizumab is a complete, humanized monoclonal antibody directed against all isoforms of VEGF. It was ﬁrst approved by the US Food and Drug Administration as a ﬁrst-line treatment for metastatic colorectal cancer. Recently, the application of intravitreal bevacizumab for the effective antiangiogenic treatment of retinal and choroidal neovascularization has been widely reported. [62]. In this study, we chose bevacizumab as a positive control in the in vitro studies.

Although various HGF variants have been reported to inhibit in vitro and in vivo angiogenesis, we showed here for the first time that a peptide derived from HGF can inhibit VEGF-induced in vitro and in vivo angiogenesis. H-RN and H-FT have the same length of amino acid sequences; however, they have significantly different biologic effects on angiogenesis. We demonstrated that H-RN effectively inhibited VEGF-induced choroid-retinal endothelial cell proliferation, migration, and tube formation, as well as in vivo angiogenesis in CAM and OIR in C57BL/6J mice. H-FT and scrambled peptide had no such effects. In the in vitro studies, H-RN exhibited more potency in inhibiting endothelial cell migration than inhibiting cell proliferation and tube formation; the effective concentrations were much lower than those in the migration and tube formation assays. Perhaps inhibiting endothelial cell migration is one of the critical mechanisms of H-RN antiangiogenesis activity.

Xin et al. [49] first reported in 2000 that kringle 1 of HGF effectively inhibited endothelial cell proliferation. In subsequent studies, they showed that the same kringle structure could also inhibit endothelial cells’ capillary-like tube formation, tumor angiogenesis, and tumor growth [50]. Kringle 1 of HGF is the mother domain of the peptides in our present study, and although H-RN has no such strong antiproliferative effect, it can effectively inhibit endothelial cell migration, tube formation, and angiogenesis in vivo. Hence, the sequence of H-RN studied might be a part of the critically active sequence of HGF kringle 1 in antiangiogenesis.

Mayo et al. [63] reported that aliphatic hydrophobic residues are commonly seen in peptides with antiangiogenic activity, and these hydrophobic residues are all in proximity to each other. With this study, we have gained an insight into two peptides derived from HGF K1, H-RN, which was shown to inhibit angiogenesis effectively, does have more hydrophobic residues than H-FT, which was inactive in antiangiogenesis. In addition, the hydrophobic residues in the H-RN sequence were much more in proximity to each other than those in H-FT sequence. This was in accordance with Mayo’s discovery, and also provides additional evidence for the phenomenon that proximal hydrophobic residues are relatively commonly seen in peptides with antiangiogenic activity.

RF/6A, which is a choroid-retinal endothelial cell line, is widely used for in vitro studies on retinal and choroidal neovascularization [56,64]. As was shown in this study, H-RN could effectively inhibit RF/6A cell proliferation, migration, and tube formation, which indicates its potential for inhibiting retinal and choroidal neovascularization. This was demonstrated by in vivo retinal neovascularization in the mouse model of OIR. VEGFab is a neutralizing VEGF antibody that can block the bioactivity of mouse VEGF. The reason we chose VEGFab instead of bevacizumab to be a positive control in the OIR mouse model was that bevacizumab is a humanized variant of the antihuman VEGF mAb. It has been characterized as species-specific, and has little or no interaction with murine VEGF [65].

In this 17-day model, treatment with H-RN of different concentrations on day 12 and day 14 controlled the degree of blood vessel tuft formation and blood vessel tortuosity, though not as effectively as did VEGFab. Even at 10 mM, H-RN appeared to be an active inhibitor of neovacularization in OIR, which inhibited neovascular lumens by 50%. Furthermore, this peptide improved retinopathy by abrogating the invasion of new vessels beyond the inner-limiting membrane of the retina, according to the analysis of eye sections. The CAM model is widely used to study angiogenic phenomena because of its easy access to the blood vessel network, its function in low immunocompetence, and the time of this assay is relatively short [66]. In the present study, H-RN was found to effectively inhibit CAM angiogenesis. This might be considered a sign of the possible antiangiogenic activity of H-RN in other cases of neovascularization. We may investigate this in further studies.

H-RN demonstrated significant stability in different aqueous solutions. After 48 h of incubation in 37 °C solutions, less than 5% of the H-RN degraded. It was even more stable than an acylated 28-amino acid peptide described by Staes et al. [54]. BSS PLUS™ has no components foreign to the eye and no pharmacological action. It contains the appropriate bicarbonate, pH, and ionic composition necessary for the maintenance of normal retinal electrical activity. This is why it was chosen as a test solution in the stability assay. However, the actual intraocular condition is more complicated, including various proteases, inflammatory cells, and cytokines caused by an injection procedure or neovascularization disease. Hence, further investigation of H-RN stability in vivo is needed in the future.

A key finding in this study is that H-RN is a newly discovered peptide derived from HGF. Its antiangiogenic activity is not as strong as that of VEGF-neutralizing antibodies, but it is easy and inexpensive to synthesize, and it possesses the other advantages of small peptides described above. However, there are still some limitations to the use of peptides as drugs. They have a short half-life and low stability in vivo, due to their degradation by proteases. There are some other disadvantages of large peptides, such as immune response induction, poor bioavailability, limited delivery by injection, though small peptides that have fewer than 20 amino acids could have those effects to a less degree [21].

H-RN is a small peptide with 11 amino acids. In the OIR model, its effective concentration reached the millimolar scale, and reinjection problems could exist in future research. Some chemical modifications would be helpful for increasing its half-life, such as substituting D-amino acids for L-amino acids, cyclization, substitution by noncoded amino acids, end modifications, and modification of peptide side chain. The addition of sugars or salts, as well as heparin, can enhance the thermal stability of peptides, cause self-association, and modulate solubility. Non-ionic surfactants stabilize peptides against self-aggregation. Cationic and anionic surfactants facilitate absorption of peptides across biologic membranes. Protease inhibitors, such as sodium glycocholate, can also be coadministered with the therapeutic agent. New drug delivery systems, such as intravitreal slow-release delivery systems, should be developed to achieve an effective drug concentration in the retina or choroidal tissues [67]. These efforts might help decrease the necessary concentration of H-RN.

In the present study, all the above experiments primarily demonstrated the antiangiogenic activity of H-RN. In future studies, we will investigate the pharmacodynamics and pharmacokinetics of H-RN, modified synthetic H-RN with improved pharmacodynamics and pharmacokinetics, the use of endothelial cell-specific cell penetrating peptides, the controlled-release carrier or lentiviral vector as drug carriers, and novel delivery methods, as described in a review paper [68]. These works will promote the development of novel peptide drugs and clinical applications.

From the lines of evidence described above, a new peptide derived from HGF was shown to have antiangiogenic activity in a choroid-retinal endothelial cell line, in the chick chorioallantoic membrane, and in a mouse model of oxygen-induced retinopathy. Retinal and choroidal neovascularization occurring as a severe complication of diabetes mellitus, as age-related macular degeneration, and as ROP can cause vision impairment and blindness. The identification of novel small peptides such as H-RN that can effectively inhibit retinal neovascularization may lead to new potential drug discoveries and the development of new treatments for pathological retinal angiogenesis.
